# Mechanisms of JARID1B Up-Regulation and Its Role in *Helicobacter pylori*-Induced Gastric Carcinogenesis

**DOI:** 10.3389/fonc.2021.757497

**Published:** 2021-10-28

**Authors:** Lixin Zheng, Yujiao Wu, Li Shen, Xiuming Liang, Zongcheng Yang, Shuyan Li, Tongyu Li, Wenjing Shang, Wei Shao, Yue Wang, Fen Liu, Lin Ma, Jihui Jia

**Affiliations:** ^1^ Key Laboratory of Experimental Teratology, School of Basic Medical Sciences, Shandong University, Jinan, China; ^2^ Shandong Provincial Key Laboratory of Infection and Immunology, School of Basic Medical Sciences, Shandong University, Jinan, China

**Keywords:** gastric cancer, *Helicobacter pylori*, JARID1B, miR-29c, carcinogenesis

## Abstract

Gastric cancer (GC) is the third leading cause of cancer-related death worldwide. *Helicobacter pylori* infection can induce GC through a serial cascade of events, with emerging evidence suggesting the important role of epigenetic alterations in the development and progression of the disease. Here, we report on mechanisms responsible for Jumonji AT-rich interactive domain1B (JARID1B) upregulation in GC and its role in the malignant transformation induced by *H. pylori* infection. We found that upregulation of JARID1B was associated with poorer prognosis, greater tumor purity, and less immune cell infiltration into the tumor. Mechanistically, we showed that the upregulation of JARID1B in human GC was attributed to JARID1B amplification and its induction by *H. pylori* infection. Furthermore, we identified miR-29c as a negative regulator of JARID1B in GC. *H. pylori* caused downregulation of miR-29c in human GC and thereby contributed to JARID1B upregulation through relieving posttranscriptional regulation. Functionally, we showed that knockdown of JARID1B reduced GC cell proliferation induced by *H. pylori* infection. Subsequently, cyclinD1 (CCND1), a key molecule in GC, was shown to be a target gene of JARID1B. In conclusion, these results suggest that JARID1B may be an oncogene upregulated in human GC and could represent a novel therapeutic target to prevent malignant transformation induced by *H. pylori* infection.

## Introduction

Gastric cancer (GC) is one of the most common malignancies worldwide, with >950,000 newly diagnosed cases each year (1). Due to the lack of early diagnostic screening, most patients are diagnosed at an advanced stage (2). Therefore, studying the etiology of GC is of great significance for identifying means of early detection and/or prevention.

Gastric carcinogenesis is a complex, multistep, and multifactorial event. *Helicobacter pylori* infection has long been recognized as a Class I oncogenic factor in GC. *H. pylori* can cause chronic gastritis, intestinal metaplasia, and even dysplasia, which can be considered precancerous lesions. Hence, eradication of *H. pylori* infection can reduce the risk of GC (3, 4). Cytotoxin-associated gene A (CagA) is the main virulence factor of *H. pylori* that could be injected into host cells by type IV secretory system (5). As a pathogenic factor, *H. pylori* can induce host genomic instability, including abnormal DNA methylation and disorders of micro-RNA expression (6–8). However, the exact carcinogenic mechanisms responsible for GC induced by *H. pylori* have not yet been fully elucidated.

Growing evidence is suggesting that epigenetic modifications, including histone demethylation, play important roles in the development of human tumors (9). It has been reported that histone demethylases such as those of the KDM1 [lysine (K) demethylase 1] family and Jumonji C (JmjC) domain-containing family are related to carcinogenesis (10). For example, lysine specific demethylase 1 (LSD1) is abnormally expressed in breast cancer, lung cancer, colon cancer, and GC and is involved in the regulation of tumor development (10–12). Our group has also reported that JMJD2B promotes GC cell proliferation and metastasis through cooperation with β-catenin (13, 14). JARID1B (also termed KDM5B) is a member of the JMJC histone demethylase family and was shown to be involved in tumor progression and metastasis, including GC (15–20). However, the mechanism of JARID1B upregulation in GC remains unclear.

Given the possible pathogenetic mechanisms of GC, some of the histone demethylases induced by *H. pylori* infection might contribute to its tumorigenesis and progression (13, 21, 22). Our group has reported that *H. pylori* can promote the development of GC by enhancing the expression of JMJD2B (13). Overexpression of the *H. pylori*-induced histone demethylase PHF8 exacerbated metastasis and progression of GC *via* regulation of vimentin (23). However, the role of JARID1B in the malignant transformation induced by *H. pylori* is unclear, and the underlying mechanisms remain to be elucidated. In the present study, we evaluated the clinical significance of JARID1B overexpression. For the first time, we investigated the expression, functions, and molecular mechanisms of JARID1B for *H. pylori-*induced GC progression in a series of *in vitro* and *in vivo* assays.

## Materials and Methods

### Cell Culture

The human gastric adenocarcinoma GES-1, BGC-823, MGC-801, SGC-7901, and HGC-27 cell lines were cultured in RPMI-1640 medium (Gibco, Carlsbad, CA, USA) supplemented with 10% fetal bovine serum (FBS), except for AGS in F12 medium (HyClone, USA) supplemented with 10% FBS (Gibco).

### Clinical Samples

Fourteen RNA samples were harvested from the GC patients undergoing surgical resection in Shandong Tumor Hospital. Atrophic gastritis (AG) specimens were from 63 patients with or without *H. pylori* infection of Jinan Central Hospital. The 13C urea breath test was used to detect the *H. pylori*. Histological typing of the tumors was used to confirm the diagnosis.

### *H. pylori* Cultures and *H. pylori*-Infected Mice

Three *H. pylori* strains were cultured in Brucella broth containing 5% FBS at 37°C for 48 h under microaerophilic conditions. The bacteria were added to cells with the multiplicity of infection (MOI) at 100. C57BL/6 mice (male) were separated into three groups. Among them, group 1 is the control group. Groups 2 and 3 were first given the N-methyl-N-nitrosourea (MNU, 30 ppm) in the drinking water; 10 weeks later, they were given distilled water for another 2 weeks. Thereafter, Group 3 was given the SS1 strain (1 × 10^9^ colony-forming units (CFU)/ml) every other day for three times. Group 1 was provided with distilled water without MNU or SS1. Finally, all mice were sacrificed after 50 weeks.

### Transfection

JARID1B siRNAs (Sigma-Aldrich, USA), miR-29c mimic, and inhibitor (Ribobio, Guangzhou, China) transfection were performed with the Lipofectamine 2000 (Invitrogen, Carlsbad, CA, USA). Sequences for these siRNAs: 5′-CAUAUUCUCUCUUAGAGGUdTdT-3′ (JARID1B si1) and 5′ -CAGUGAAUGAGCUCCGGCAdTdT-3′ (JARID1B si2).

### Colony Formation Assay

Cells (600 cells/well) were seeded into the plates after respective treatments, then they were incubated for about 8–14 days until colonies appeared. The colonies were then treated with methanol and Giemsa. The experiments were performed three times.

### EdU Staining

DNA synthesis was assessed by the Cell-Light EdU (5-ethynyl-2'-deoxyuridine) Apollo488 *In Vitro* Imaging Kit according to the manufacturer’s instructions (RiboBio).

### Immunohistochemistry

Paraffin-embedded sections acquired from samples were deparaffinized and rehydrated. Then, the citric acid buffer was used for antigen retrieval. Next, after H_2_O_2_ treatment and nonspecific antigen blocking for 30 min, sections were incubated with the primary JARID1B antibody (1:100, ab181089, Abcam, Cambridge, UK). Next day, the colorimetric detection was performed using DAB (3,3'-diaminobenzidine) staining kit (Vector Laboratories, USA).

### RNA Extraction and RT-PCR

Total RNA was extracted with TRIzol reagent (Invitrogen, Carlsbad, CA, USA) as per the manufacturer’s instructions. RNAs were then reverse-transcribed by the PrimeScript RT reagent Kit with gDNA Eraser (Takara, Japan). Finally, SYBR-Green (TaKaRa, Japan) was then used for the real-time PCR analysis. The primers used in this real-time quantitative PCR (RT-qPCR) assay are as follows: 5′-TGTCACAGTGGAATATGGAGCTGAC-3′ and 5′-GCCACTATCAAGATACTCCTCTTCC-3′ for JARID1B; 5′-ATGGAACACCAGCTCCTGTG-3′ and 5′-ACCTCCAGCATCCAGGTGGC-3′ for CCND1; 5′-AGTTGCGTTACACCCTTTCTTG-3′ and 5′-CACCTTCACCGTTCCAGTTTT-3′ for β-actin; 5′-GAATTGCTATGTGTCTGGGT-3′ and 5′-CATCTTCAAACCTCCATGATG-3′ for β2-M.

### Western Blotting

Cells were lysed with the protein lysis buffer supplemented with protease inhibitors for 30 min on ice to extract the protein. Next, proteins were subjected through sodium dodecyl sulfate–polyacrylamide gel electrophoresis (SDS-PAGE) and transferred to the polyvinylidene fluoride (PVDF) membranes. Then, 5% nonfat milk was used to block the membranes. The specific primary antibodies and the respective secondary antibodies were also used for incubation. Immunoblots were visualized with ECL (Enhanced chemiluminescent) reagent (Millipore). The primary antibodies were specific for JARID1B (Abcam), β-actin (Sigma-Aldrich, St. Louis, MO, USA), CCND1 (Cell Signaling, Danvers, MA, USA), and CagA (Santa Cruz Biotechnology, Santa Cruz, CA, USA).

### Luciferase Reporter Assay

The human JARID1B 3′-untranslated region (UTR) and corresponding mutants were cloned into the pMIR-GLO basic luciferase reporter vector (Promega, Madison, WI, USA). GC cells were transfected with the internal control vector pRL-TK and the promoter or 3′-UTR reporters using Roche Transfection Reagent (Roche, Basel, Switzerland) 24 h after seeding. Luciferase reporter activity was measured by a Luciferase Assay System (Promega) as per the manufacturer’s instructions 48 h after transfection.

### Chromatin Immunoprecipitation Assay

For chromatin immunoprecipitation (ChIP) assay, the SimpleChIP^®^ Enzymatic Chromatin IP Kit (Cell Signaling, Danvers, MA, USA) was used according to the manufacturer’s protocol. The precipitated DNA samples were detected with PCR method.

### Identification of Upregulated Differentially Expressed Genes

The original files (.CEL files) and platform files of the GSE54129 dataset in the Gene Expression Omnibus (GEO) database (http://www.ncbi.nlm.nih.gov/geo/) were downloaded. The raw data files were analyzed using the Robust Multi-array Average algorithm with the “affy” package and the “impute” package in R/Bioconductor software (version 3.5.3). For the upregulated differentially expressed genes (DEGs), we used the “limma” package and set P < 0.05. The inside-software plugin cytoHubba was used to identify hub genes among the genes of interest. This program provides a user-friendly interface to explore important nodes in biological networks and computes using 11 methods (24). Metascape is a free online resource with an automated meta-analysis tool for understanding the biological significance of a large number of genes and can identify enriched pathways and be used for the construction of protein–protein interaction networks from lists of gene and protein identifiers (25).

### Statistical Analysis

All data are presented as means ± standard deviations from three independent assays. The Student’s t-test or Mann–Whitney U test was used to determine the significance of differences in each two-group comparison. Statistical analysis was performed using SPSS version 23.0. Correlation analysis of the data was performed using linear regression. Differences were considered statistically significant when P < 0.05.

## Results

### Increased JARID1B Expression Predicts Poorer Clinical Outcome in Gastric Cancer Patients

**Figure 1:**
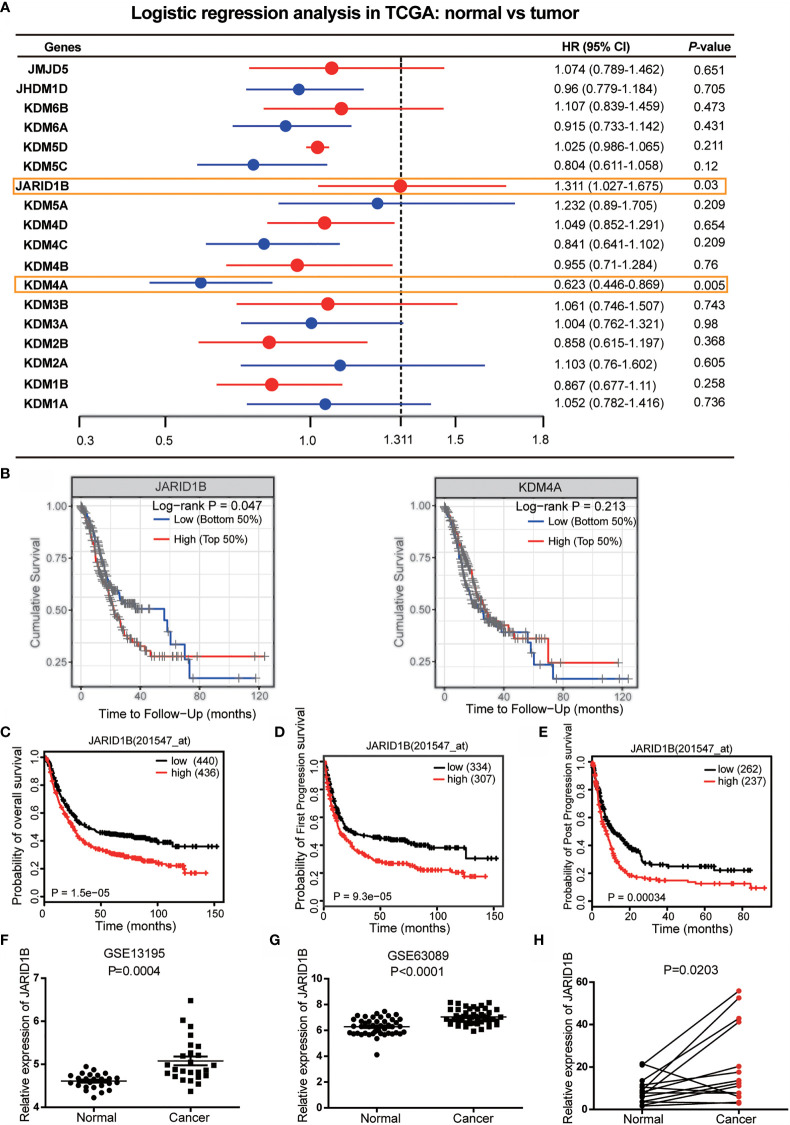
JARID1B overexpression in gastric cancer (GC) patients is associated with poorer prognosis. **(A)** The logistic regression analysis of 18 histone demethylase gene for the prognosis of GC in The Cancer Genome Atlas (TCGA) database. **(B)** Further analysis of the prognostic significance of JARID1B and KDM4A in GC patients. **(C–E)** Relationship between JARID1B expression and overall survival **(C)**, first progression survival **(D)**, the post progression survival **(E)** in GC patients (log-rank test; n = 876), both data and P values are from the Kaplan–Meier Plotter database. **(F, G)** GEO RNA sequencing database analysis of the relatively differential expression levels of JARID1B in human GC and paired adjacent normal tissues; the data were derived from GSE13195 **(F)** and GSE63089 **(G)**. **(H)** The messenger RNA (mRNA) levels of JARID1B in 14 GC and paired adjacent normal tissues were measured by real-time PCR (n = 14).

To identify potential roles of histone demethylases in human GC pathogenesis, we first undertook logistic regression analysis of 18 histone demethylase genes to establish hazard ratio (HR) values for their influence on GC prognosis using data from The Cancer Genome Atlas (TCGA) database. This approach showed that JARID1B and KDM4A (Lysine-specific demethylase 4A) were statistically significant (**Figure 1A**). Further analysis indicated that only JARID1B had a significant prognostic value in GC patients (**Figures 1B** and **S1**). Additionally, we performed a bioinformatics analysis of JARID1B expression profiles from 876 GC patients using the Kaplan–Meier method and found that high expression of JARID1B was significantly correlated with poorer overall survival, first progression survival, and post-progression survival of GC patients (**Figures 1C–E**). Furthermore, we also carried out an analysis of the GEO databases (e.g., GSE27342 and GSE63089), which showed that JARID1B expression was upregulated in GC tissues (**Figures 1F, G**). Analysis of 14 GC biopsies and their corresponding adjacent non-tumor tissues confirmed this result (**Figure 1H**). Similarly, JARID1B was also differentially expressed in several gastric cell lines, including GES-1, AGS, BGC-823, HGC-27, MGC-803, and SGC-7901 (**Figure S2**). Taken together, the data imply an oncogenic role for histone demethylase JARID1B in GC.

### High Expression of JARID1B Is Associated With Greater Tumor Purity and Less Immune Cell Infiltration in Gastric Cancer

**Figure 2:**
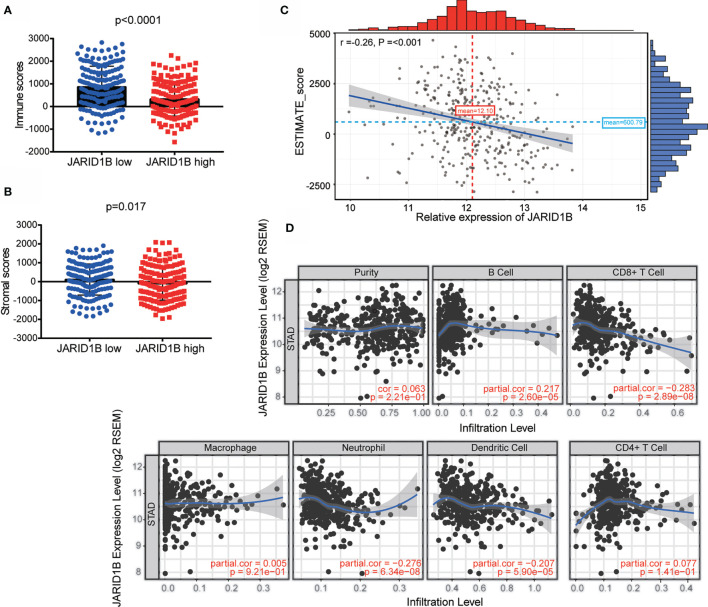
JARID1B overexpression is associated with greater tumor purity and immune infiltration of gastric cancer (GC). **(A, B)** Relationship between JARID1B expression and the immune score, stromal score group in TIMER of GC. **(C)** Correlation between JARID1B expression and ESTIMATE score in TIMER of GC. **(D)** Correlation between JARID1B expression and CD8+ T cells, neutrophils, as well as dendritic cells in TIMER of GC.

The tumor microenvironment consists of tumor cells, stromal cells, and infiltrating immune cells, and it has been proposed that these variables are valuable for diagnostic and prognostic assessment of tumors. We therefore utilized TIMER (Tumor IMmune Estimation Resource) to assess the role of JARID1B in GC by adopting the ESTIMATE algorithm (from https://bioinformatics.mdanderson.org/estimate/). This analysis showed that the level of JARID1B messenger RNA (mRNA) was significantly higher in the low immune score and low stromal score group (**Figures 2A, B**). Furthermore, high expression of JARID1B was statistically significantly associated with immune score (low, P < 0.0001) and stromal score (low, P = 0.017; **Figures 2A, B**). The expression of JARID1B was negatively correlated with the ESTIMATE score (r = -0.26, P <0.001; **Figure 2C**), suggesting that JARID1B was positively associated with tumor purity of GC. Moreover, analysis of tumor-infiltrating immune cells showed that high expression of JARID1B was negatively associated with the amount of CD8+ T cells, neutrophils, and dendritic cells in the tumor (**Figure 2D**).

### Gene Amplification of JARID1B in Gastric Cancer

**Figure 3:**
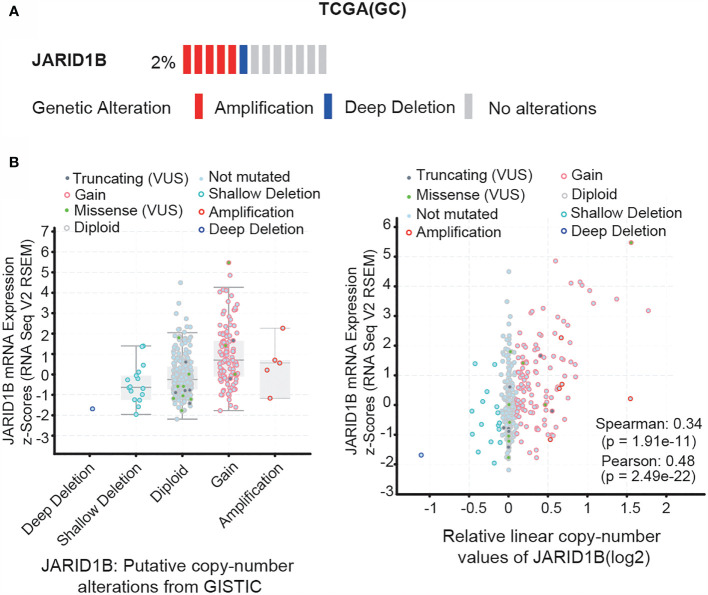
Gene amplification of JARID1B in gastric cancer (GC). **(A)** cBioPortal (http://www.cbioportal.org/) analysis of the JARID1B genetic amplification in The Cancer Gene Atlas (TCGA) GC tissues. **(B)** Correlation between JARID1B copy number alteration and the messenger RNA (mRNA) expression.

Next, we investigated the mechanism responsible for JARID1B upregulation in GC. We searched for information on JARID1B gene mutations, as well as the inherent connection between gene copy number and mRNA expression in the “Stomach Adenocarcinoma (TCGA, Provisional)” dataset *via* the cBioportal program (http://www.cbioportal.org). Notably, gene amplification of JARID1B was frequently found in TCGA GC tissues, and the copy number significantly correlated with levels of JARID1B mRNA expression (**Figures 3A, B**). This suggests that copy number variation may underlie JARID1B amplification in GC.

### *H. pylori* Promotes the Expression of JARID1B

**Figure 4:**
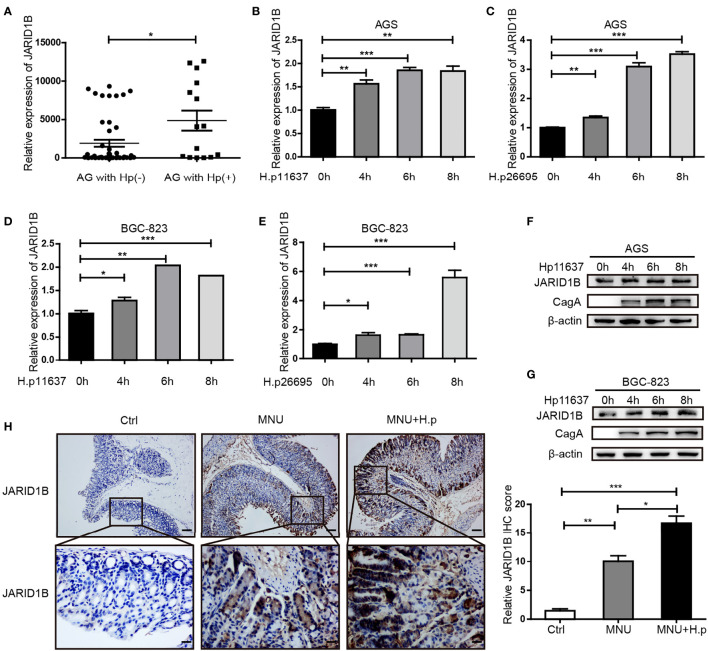
*H. pylori* promotes the expression of JARID1B in gastric cancer (GC). **(A)** JARID1B expression was analyzed in gastritis tissue with *H. pylori* positive or negative by RT-qPCR. **(B–E)** Real-time quantitative PCR (RT-qPCR) was used to detect JARID1B messenger RNA (mRNA) in AGS and BGC-823 cell lines treated with different strains of *H. pylori* at different time points, *P < 0.05, **P < 0.01, ***P < 0.001 by Student’s t-test. **(F, G)** Western blot was used to detect the protein levels of JARID1B in AGS and BGC-823 cells incubated with *H. pylori* strains (11637) at different time points. **(H)** Immunohistochemical staining analysis of JARID1B expression in gastric tissue of control group, low-dose MNU gavage group, and *H. pylori* (SSI) combined with low-dose MNU gavage group. Representative images are shown here (magnification: ×100, ×400; scale bars: 200 and 50 μm).

*H. pylori* infection is a risk factor for GC. Hence, we investigated whether *H. pylori* infection influences the abnormal expression of JARID1B in GC. We first analyzed JARID1B expression in *H. pylori*-infected gastritis tissue by RT-qPCR and documented its significant upregulation relative to *H. pylori*-positive gastritis tissues (**Figure 4A**). To further investigate the regulation of JARID1B expression by *H. pylori* infection, we used different *H. pylori* strains (*H.p11637* and *H.p26695*) to infect GC cells (AGS and BGC-823) at different time points. RT-qPCR showed that *H. pylori* infection can upregulate the expression of JARID1B mRNA at different time points (**Figures 4B–E**). Furthermore, Western blotting confirmed the upregulation of JARID1B in *H. pylori-*infected GC cells at the protein level (**Figures 4F, G** and **S3**).

*H. pylori* gavage can promote the progression of precancerous lesions in murine gastric tissue (26). To further investigate the regulation of JARID1B expression by *H. pylori* infection *in vivo*, we constructed MNU-treated mice with *H. pylori* infection. Immunohistochemistry (IHC) showed that JARID1B was slightly upregulated in the low-dose MNU group compared with the normal gastric mucosa in the control group but was significantly upregulated when low-dose MNU was combined with *H. pylori* infection (**Figure 4H**). These findings indicate that *H. pylori* promotes the expression of JARID1B both *in vivo* and *in vitro*.

### *H. pylori* Upregulates the Expression of JARID1B Through the Downregulation of miR-29c

**Figure 5:**
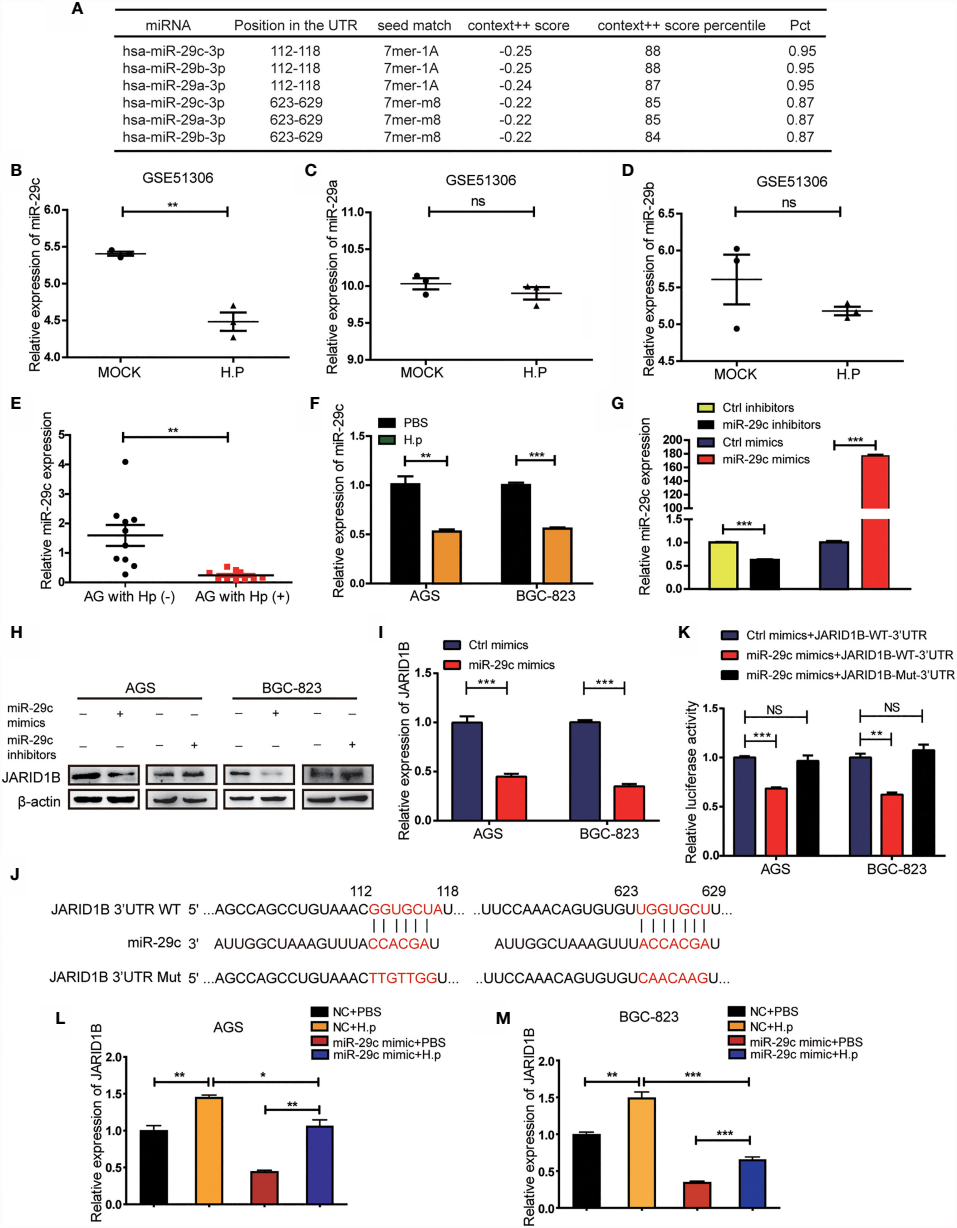
*H. pylori* upregulates JARID1B expression through the downregulation of miR-29c. **(A)** TargetScan analysis of the microRNA that can bind to 3′-untranslated region (UTR) of JARID1B. **(B–D**) The expression of miR-29c, miR-29a, and miR-29b in gastric epithelial cells infected with *H. pylori*, data came from GSE51306. **(E)** RT-PCR detection of miR-29c in gastritis tissues with or without *H. pylori* infection. **(F)** RT-PCR analysis of miR-29c expression in *H. pylori*-infected BGC-823 and AGS. **(G)** Transfection efficiency of transfected miR-29c inhibitors and mimics in GC cells AGS. **(H)** Western blot assay was performed to detect the expression of JARID1B protein in AGS and BGC-823 cells after transfection of miR-29c mimics and miR-29c inhibitor, respectively. **(I)** RT-PCR was used to detect the expression of JARID1B messenger RNA (mRNA) after transfection of miR-29c mimics in AGS and BGC-823. **(J)** The construction of the plasmid with miR-29c binding site [JARID1B-3’UTR-wild type (WT)] or the binding site mutation plasmid (JARID1B-3′-UTR-Mut). **(K)** Dual luciferase assay was performed to detect AGS and BGC-823 transfected with the miR-29c mimics and then transfected with the WT JARID1B 3′-UTR reporter plasmid or the mutant JARID1B 3′-UTR Mut reporter plasmid. **(L, M)** RT-PCR was used to detect JARID1B expression in AGS and BGC-823 of the control group, *H. pylori* infection group, and *H. pylori* infection combined with transfection of the miR-29c mimics group. All results were repeated three times. *P < 0.05; **P < 0.01; ***P < 0.001, NS, not statistically significant (Student’s t-test).

We further explored the mechanisms by which *H. pylori* regulates the expression of JARID1B, focusing on miRNAs, which are important posttranscriptional regulators. Abnormal expression of multiple miRNAs is a known result of *H. pylori* infection (27, 28). To investigate the potential role of miRNAs in the regulation of JARID1B, we first screened candidates using TargetScan and found that miR-29c, miR-29b, and miR-29a can bind to the 3′-UTR of JARID1B (**Figure 5A**). Further analysis of the GEO database (GSE51306) revealed that the expression of miR-29c was downregulated in gastric epithelial cells infected with *H. pylori*, while the expression of miR-29a and miR-29b was not significantly affected (**Figures 5B–D**). Furthermore, RT-qPCR showed that miR-29c expression in *H. pylori*-infected gastritis tissues was significantly lower than that in uninfected tissues (**Figure 5E**). In addition, miR-29c expression was downregulated in *H. pylori*-infected AGS and BGC-823 cell lines (**Figure 5F**).

To determine whether miR-29c regulates JARID1B expression, we transfected these cell lines with miR-29c analogs (mimics) and inhibitors (**Figure 5G**). Western blotting confirmed that the former decreased the amount of JARID1B protein, while the latter increased it (**Figures 5H** and **S4**). RT-qPCR showed that transfection of AGS and BGC-823 cells with miR-29c analogs significantly inhibited JARID1B mRNA expression (**Figure 5I**). To further investigate whether JARID1B is a direct target of miR-29c, we constructed a fluorescent reporter plasmid containing the JRID1B 3′-UTR of the miR-29c binding site [JARID1B-wild type (WT)-3′-UTR] and a miR-29c binding site-mutated reporter plasmid (JARID1B-Mut-3′-UTR) (**Figure 5J**). We found that the miR-29c analogs with JARID1B-WT-3′-UTR reduced the luciferase activity, which was not the case for JARID1B-Mut-3′-UTR (**Figure 5K**). In addition, *H. pylori* infection promoted the expression of JARID1B in AGS and BGC-823 cells, while JARID1B expression was downregulated when cells were transfected with miR-29c analogs (**Figures 5L, M**). These results indicate that *H. pylori* regulates the expression of JARID1B through miR-29c.

### *H. pylori* Promotes Proliferation of Gastric Cancer Cells Through JARID1B

**Figure 6:**
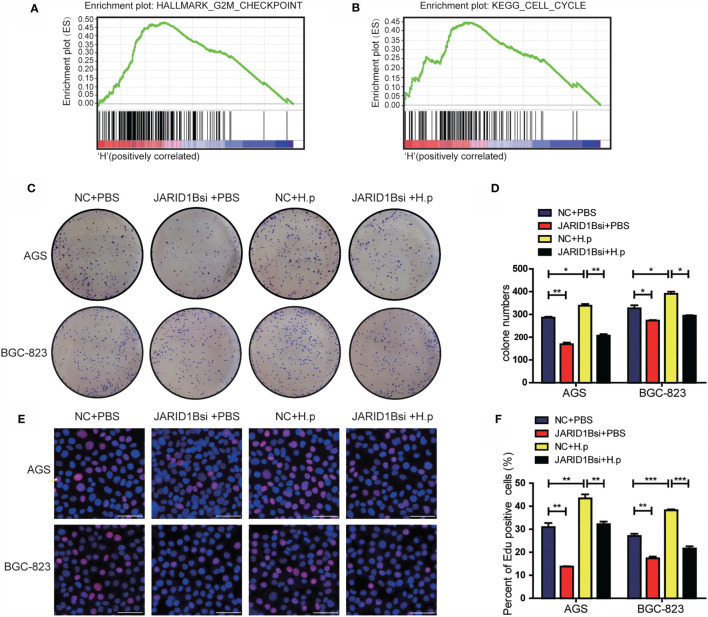
*H. pylori* promotes the proliferation of gastric cancer (GC) cells *via* JARID1B. **(A, B)** Enrichment plots from gene set enrichment analysis (GSEA). **(C, D)** Cloning formation test including the control group, JARID1B siRNA group, *H. pylori* infection group, and JARID1B siRNA combined with *H. pylori* infection group in AGS and BGC-823 cells, representative picture **(C)** and quantitative map **(D)**. **(E, F)** EdU assay including the control group, JARID1B siRNA group, *H. pylori* infection group, and JARID1B siRNA combined with *H. pylori* infection group in AGS and BGC-823, representative picture **(E)** and quantified map **(F)**. All results were repeated three times, and each horizontal line in the quantified graph of the figure represents the mean ± standard error of three experiments. Scale bar, 50 μm. *P < 0.05; **P < 0.01; ***P < 0.001 (Student’s t-test).

To gain a deeper understanding of the effects of JARID1B, we performed gene set enrichment analysis (GSEA) on the microarray data from TCGA database. This revealed that high JARID1B expression was positively associated with the G2M checkpoint and cell cycle control in GC (**Figures 6A, B**). As JARID1B can promote the proliferation of GC cells (**Figure S5**), to further explore the role of JARID1B in the proliferation induced by *H. pylori*, we performed colony formation and EdU staining experiments. We showed that the colony-forming capacity of GC cells in which JARID1B expression was inhibited was decreased relative to controls and that *H. pylori* infection increased colony formation. Furthermore, *H. pylori* infection together with JARID1B interference decreased colony formation relative to cells only infected with *H. pylori* (**Figures 6C, D**). The results of the EdU staining experiment were consistent with the colony formation findings (**Figures 6E, F**). Our data thus indicate that JARID1B promotes the proliferation of GC cells and is a node molecule for GC cell proliferation induced by *H. pylori*.

### JARID1B Promotes the Expression of CCND1 in Gastric Cancer Cells

**Figure 7:**
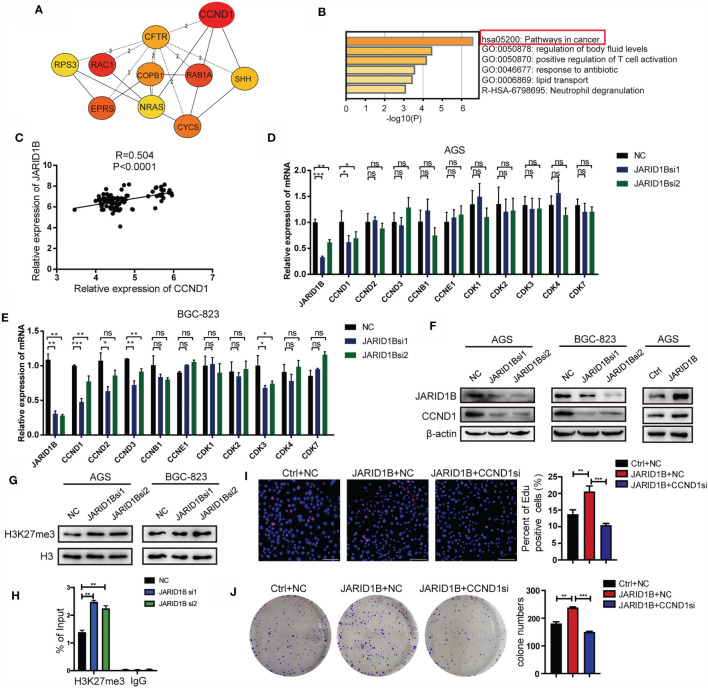
JARID1B promotes the expression of CCND1 in gastric cancer (GC) cells. **(A)** The upregulated genes that are positively related to JARID1B expression in GSE54129 of GC. **(B)** The biological function and pathway enrichment analysis with the Metascape. **(C)** The Gene Expression Omnibus (GEO) database (GSE63089) analysis showed that there was a significant positive correlation between JARID1B and CCND1 in 45 pairs of GC and corresponding adjacent normal tissues. **(D, E)** RT-PCR was used to detect the expression of proliferation-related genes in AGS and BGC-823 transfected with JARID1B siRNA. *P < 0.05; **P < 0.01; ***P < 0.001, NS, not statistically significant. (Student’s t-test). **(F)** Western blot assay was performed to detect CCND1 expression in AGS and BGC-823 after transfection with JARID1B siRNA. **(G)** The levels of H3K27me3 after JARID1B knockdown was tested by Western blot. **(H)** Chromatin immunoprecipitation (ChIP) assays were performed in JARID1B knockdown AGS cells. **(I, J)** Colony formation assays and EdU staining were used to test the role of CCND1 in the proliferation of GC cells regulated by JARID1B. Scale bar, 100 μm.

Finally, we aimed to elucidate the mechanisms by which JARID1B promotes GC cell proliferation. In this study, we analyzed the GSE54129 dataset in the GEO database. A search for upregulated DEGs positively associated with JARID1B was performed with Cytohubba. This showed that a number of genes are related to JARID1B expression, with CCND1 being the strongest (**Figure 7A**). Biological function and pathway enrichment analyses with Metascape indicated effects on six biological processes, most significantly was cancer pathways (**Figure 7B**). The analysis of 45 pairs of GC and corresponding adjacent normal tissues in the GSE63089 also revealed a significant positive correlation between JARID1B and CCND1 (**Figure 7C**). Furthermore, the expression levels of JARID1B and several proliferation-associated genes including cyclins (CCND1, CCND2, CCND3, CCNB1, and CCNE1) and cell cycle protein-dependent kinases (CDK1, CDK2, CDK3, CDK4, and CDK7) were analyzed. We observed that silencing of JARID1B decreased the expression of CCND1 in AGS and BGC-823 cells, both by RT-qPCR and Western blotting (**Figures 7D–F** and **S6**). Because JARID1B was reported to decrease H3K27me3 levels in osteosarcoma (29), we then tested the change of H3K27me3 in GC cells. The results showed that knockdown of JARID1B could enhance H3K27me3 levels in AGS and BGC-823 cells (**Figures 7G** and **S7, 8**). In addition, as H3K27me3 is associated with transcriptional repression (30), we further tested the correlation between JARID1B expression and H3K27me3 levels at the promoter of CCND1. ChIP results indicated that knockdown of JARID1B could enhance H3K27me3 levels in AGS cells (**Figure 7H**). Furthermore, the results of colony formation assays and EdU staining also showed that CCND1 is a node molecule in the proliferation of GC cells regulated by JARID1B (**Figures 7I, J**). Therefore, our data indicate that JARID1B can regulate CCND1 expression and thereby mediate the proliferation of GC cells.

## Discussion

The role of histone demethylases in tumorigenesis and progression has become more clearly recognized recently, for example, KDM4B is involved in the growth and progression of tumors such as GC, breast cancer, and ovarian cancer (31–33); HPV16 E7 and miR-342-3p stimulate the expression of KDM2A in tumors, and KDM2A then enhances ERK1/2-mediated tumor formation in lung cancer (34–36); KDM4A is abnormally expressed in colorectal cancer, prostate cancer, lung cancer, and breast cancer and promotes their progression (37, 38). It has also been reported that JARID1B is overexpressed in GC and promotes cell proliferation and metastasis (20). Here, we mainly focused on the mechanism of JARID1B upregulation in GC and report its vital role for the clinical prognosis.

Chronic *H. pylori* infection is an important risk factor for developing gastric adenocarcinoma (3). It has been reported that *H. pylori* promotes the release of inflammatory factors and the proliferation of epithelial cells by activating phosphoinositide 3-kinase (PI3K)/Akt, Wnt/β-catenin, nuclear factor (NF)-κB, and Ras signaling pathways (39–43). *H. pylori*-mediated NF-κB/miR-223-3p/ARID1A axis signaling could promote the development and progression of GC (44). Our group has demonstrated that *H. pylori* induces hepatocyte nuclear factor-4 α (HNF4α) expression *via* the NF-κB pathway (45). In the present study, analyses of clinical specimens showed that JARID1B expression was upregulated in *H. pylori*-positive gastritis tissues. Moreover, both *in vitro* and *in vivo* studies indicated that *H. pylori* infection can induce the expression of JARID1B.

miRNAs are important posttranscriptional regulators, and *H. pylori* infection may contribute to their dysregulation (46–48). Here, we found that *H. pylori* infection inhibited miR-29c expression and led to the enhancement of JARID1B by targeting its 3′-UTR region in gastric epithelial cells. In addition to the posttranscriptional regulation, transcriptional regulation also plays important roles in gene expression control. Our analysis of the JARID1B promoter region showed that transcription factors E2F1, p53, Sp1, and Sp3 are also potential regulators of JARID1B expression. Therefore, we will further explore the regulatory effect of these transcription factors on JARID1B expression and their specific mechanisms in the future.

Sustained cell proliferation is one of the hallmarks of tumorigenesis (49). Abnormal proliferation of cells is subject to complex regulatory processes accompanied by disordered expression of oncogenes or cell cycle-related genes. Many abnormally expressed genes mediate tumor development and progression by regulating cell proliferation. For example, SMYD3 promotes tumorigenicity and intrahepatic metastasis of hepatocellular carcinoma cells by regulating CDK2 and matrix metalloproteinase (MMP)2 expression (50). Both our colony formation and EDU experiments showed that JARID1B interference significantly inhibits the proliferation of GC cells induced by *H. pylori* infection. Moreover, our further analyses indicated that JARID1B downregulation prevents the expression of CCND1. Because CCND1 is a cell cycle regulatory protein that influences cell proliferation and has cancer-promoting properties in many tumors including GC (51), JARID1B may promote cell proliferation through CCND1. On the other hand, we also found that JARID1B may promote the expression of CCND1 through the regulation of H3K27me3 levels at the promoter of CCND1.

In summary, this study explored the mechanisms responsible for upregulating the histone demethylase JARID1B in GC. We first revealed that *H. pylori* infection can promote JARID1B expression in GC cells through miR-29c. High expression of JARID1B in GC patients indicates a poorer prognosis, and interference of JARID1B may attenuate the proliferation of GC cells induced by *H. pylori*. This study further contributes to our understanding of gene regulation abnormalities in the development of GC and has important implications for intervention to prevent malignant transformation induced by *H. pylori* infection.

## Data Availability Statement

The original contributions presented in the study are included in the article/**Supplementary Material**. Further inquiries can be directed to the corresponding author.

## Ethics Statement

The studies involving human participants were reviewed and approved by Shandong University Research Ethics Committee. The patients/participants provided their written informed consent to participate in this study. The animal study was reviewed and approved by Shandong University Research Ethics Committee.

## Author Contributions

JJ designed the study. LZ, YWu, LS, XL, SL, TL, WJS, WS, and YWa performed experiments. LZ, ZY, and FL analyzed data. JJ, LZ, and LM obtained funding. LZ and JJ prepared the figures. LZ and JJ wrote the manuscript. JJ supervised the study. All authors contributed to the article and approved the submitted version.

## Funding

This work was supported by the National Natural Science Foundation of China (Nos. 81772151, 81971901, 82002107, 81871620, 82172284 and 81801983) and the Department of Science and Technology of Shandong Province (No. 2018CXGC1208).

## Conflict of Interest

The authors declare that the research was conducted in the absence of any commercial or financial relationships that could be construed as a potential conflict of interest.

## Publisher’s Note

All claims expressed in this article are solely those of the authors and do not necessarily represent those of their affiliated organizations, or those of the publisher, the editors and the reviewers. Any product that may be evaluated in this article, or claim that may be made by its manufacturer, is not guaranteed or endorsed by the publisher.
