# Supplementary material for: Mechanisms of JARID1B Up-Regulation and Its Role in Helicobacter pylori-Induced Gastric Carcinogenesis
Source: Front Oncol. 2021 Oct 28;11:757497. doi: 10.3389/fonc.2021.757497 (PMC8581301; doi:10.3389/fonc.2021.757497)
Supplement: Supplementary file 1 [file DataSheet_1.pdf]

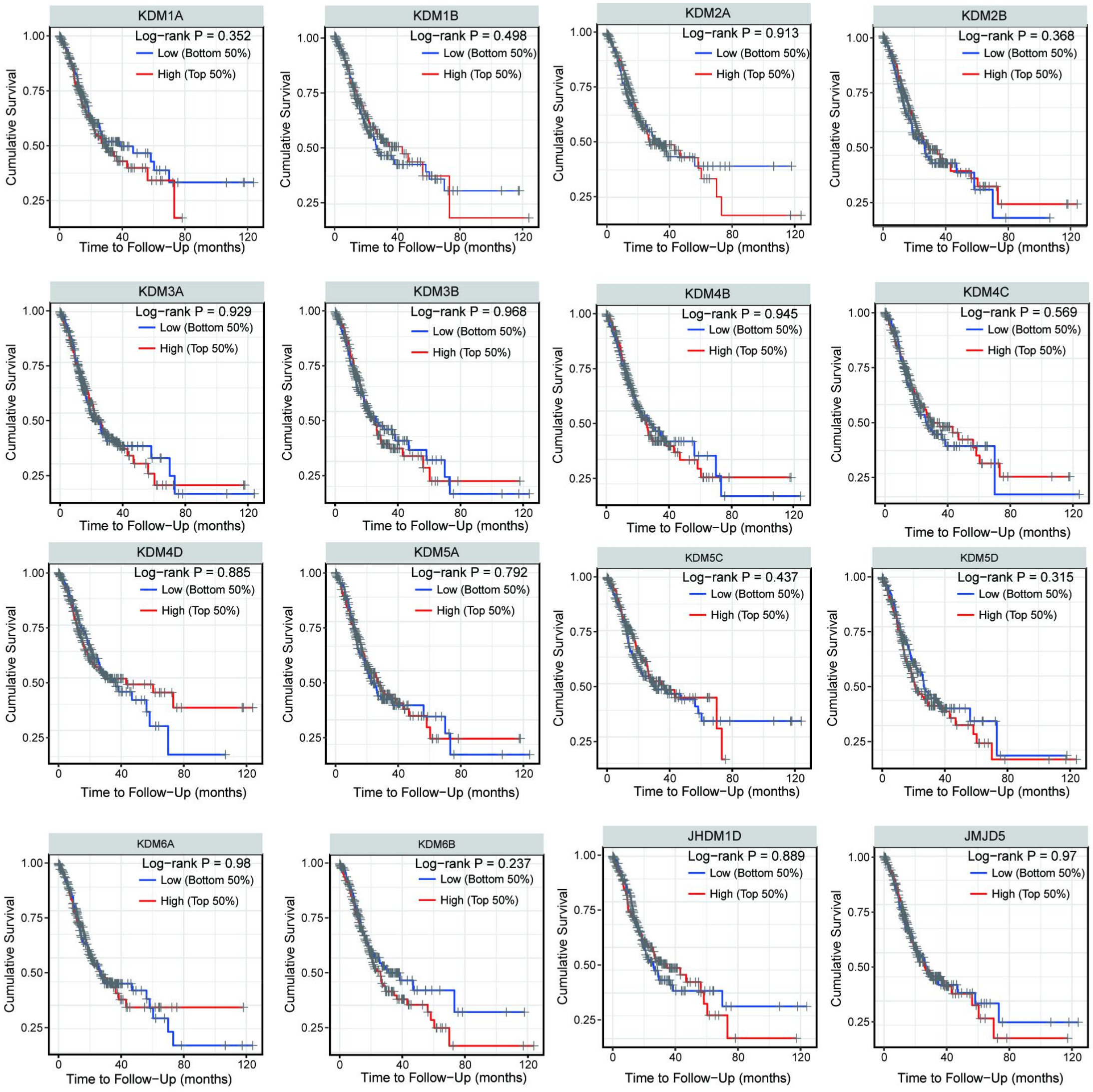


**Figure S1.** The analysis of the other 16 histone demethylase gene for the prognosis in the TCGA database GC patients.

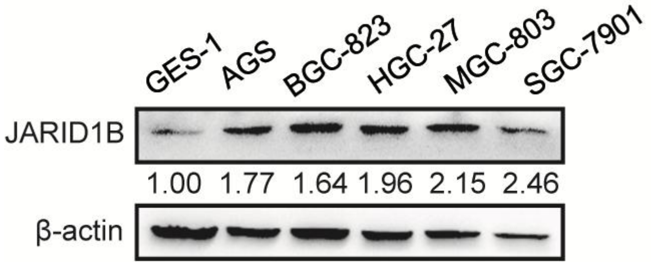


**Figure S2.** The protein levels of JARID1B in different gastric cancer cell lines and immortalized gastric cells GES-1 by Western blot.

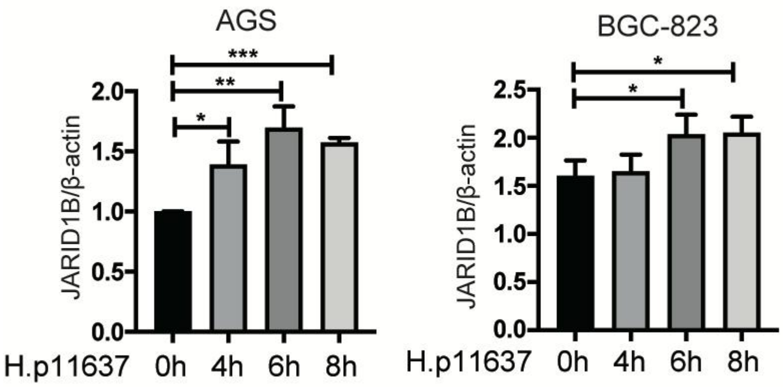


**Figure S3.** The quantitative analysis of the Western blots results (Figures 4F-4G).

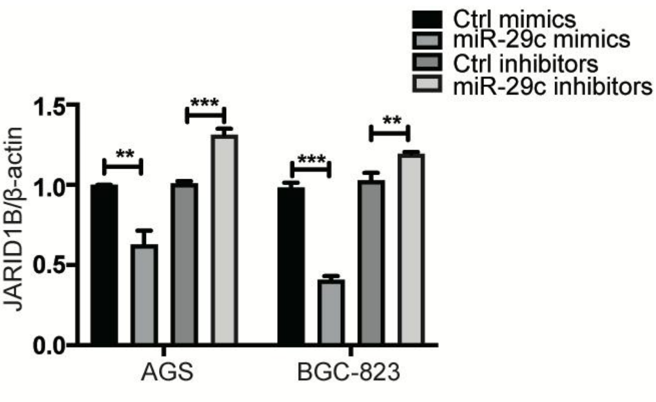


**Figure S4.** The quantitative analysis of the Western blots results (Figures 5H).

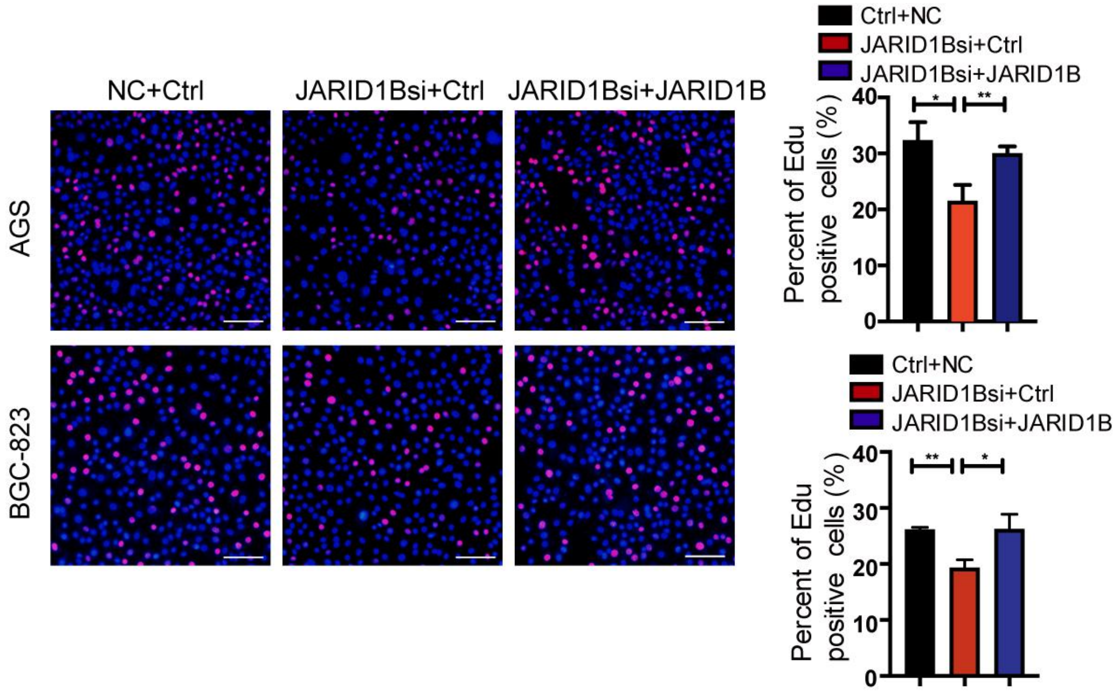


**Figure S5.** Representative images and quantification of Negative Control (NC) - or JARID1B siRNA-transfected AGS and BGC-823 cells in EdU assays. Scale bars, 100 μm.

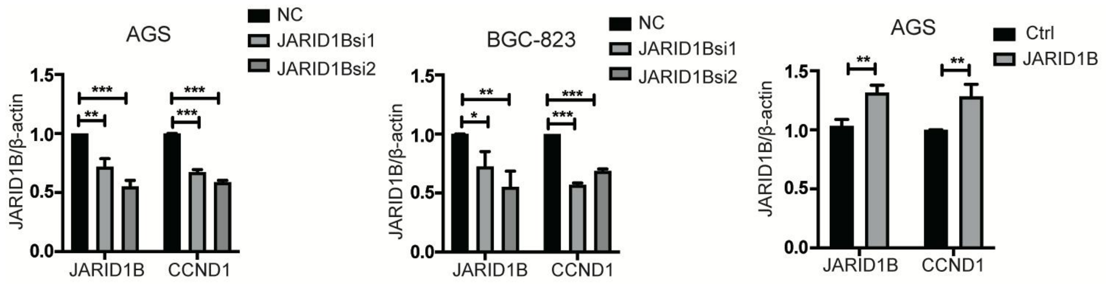


**Figure S6.** The quantitative analysis of the Western blots results (Figures 7F).

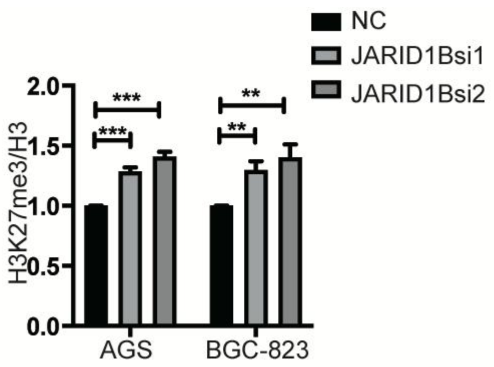


**Figure S7.** The quantitative analysis of the Western blots results (Figures 7G).

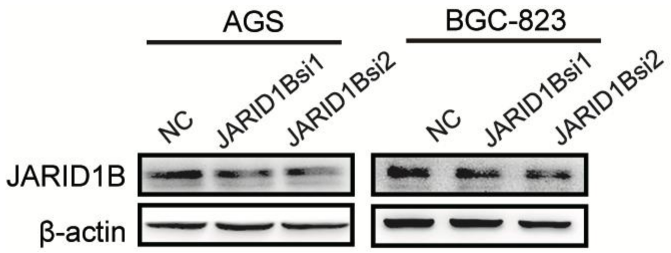


**Figure S8.** The expression of JARID1B in JARID1B knockdown AGS and BGC cells.
